# Coronary aspirate TNFα reflects saphenous vein bypass graft restenosis risk in diabetic patients

**DOI:** 10.1186/1475-2840-12-12

**Published:** 2013-01-10

**Authors:** Theodor Baars, Thomas Konorza, Philipp Kahlert, Stefan Möhlenkamp, Raimund Erbel, Gerd Heusch, Petra Kleinbongard

**Affiliations:** 1 Institut für Pathophysiologie, Universitätsklinikum Essen, Hufelandstr. 55, 45122, Essen, Germany; 2 Klinik für Kardiologie, Universitätsklinikum Essen, Universität Duisburg-Essen, Essen, Germany; 3 Krankenhaus Bethanien Moers, Moers, Germany

**Keywords:** Coronary disease, Diabetes mellitus, Ischemia, TNFα, Vasoconstriction

## Abstract

**Background:**

Patients with diabetes mellitus (DM) have an increased risk for periprocedural complications and adverse cardiac events after percutaneous coronary intervention. We addressed the potential for coronary microvascular obstruction and restenosis in patients with and without DM undergoing stenting for saphenous vein bypass graft (SVG) stenosis under protection with a distal occlusion/aspiration device.

**Methods:**

SVG plaque volume and composition were analyzed using intravascular ultrasound before stent implantation. Percent diameter stenosis was determined from quantitative coronary angiography before, immediately after and 6 months after stent implantation. Coronary aspirate was retrieved during stent implantation and divided into particulate debris and plasma. Total calcium, several vasoconstrictors, and tumor necrosis factor (TNF)α in particulate debris and coronary aspirate plasma were determined.

**Results:**

Patients with and without DM had similar plaque volume, but larger necrotic core and greater particulate debris release in patients with than without DM (20.3±2.7 *vs*. 12.7±2.6% and 143.9±19.3 *vs*. 75.1±10.4 mg, *P*<0.05). The TNFα concentration in particulate debris and coronary aspirate plasma was higher in patients with than without DM (15.9±6.6 *vs*. 5.1±2.4 pmol/mg and 2.2±0.7 *vs*. 1.1±0.2 pmol/L, *P*<0.05), whereas total calcium and vasoconstrictors were not different. Patients with DM had a greater percent diameter stenosis 6 months after stent implantation than those without DM (22.17±5.22 *vs*. 6.34±1.11%, *P*<0.05). The increase in TNFα immediately after stent implantation correlated with restenosis 6 months later (r=0.69, *P*<0.05).

**Conclusion:**

In diabetics, particulate debris and coronary aspirate plasma contained more TNFα, which might reflect the activity of the underlying atherosclerotic process.

**Trial registration:**

URL: http://www.clinicaltrials.gov/ct2/results?term=NCT01430884; unique identifier: NCT01430884

## Background

Interventional plaque rupture induces the release not only of particulate debris, but also of soluble vasoconstrictor, thrombogenic and inflammatory substances from the lesion. Both, particulate debris as well as soluble substances, contribute to impair microvascular coronary perfusion [1,2] with typical consequences: microinfarcts with a subsequent inflammatory reaction [3], arrhythmias, contractile dysfunction, and impaired coronary reserve [4]. We have previously reported the release of serotonin, thromboxane (Tx)A_2_, and tumor necrosis factor (TNF)α into the coronary aspirate retrieved from patients during stenting of stenotic saphenous vein bypass grafts (SVG) [5-8].

Diabetes mellitus (DM) is associated with a higher risk for periprocedural complications and more adverse cardiac events after percutaneous coronary interventions (PCI) [9-12], including stent implantation into SVGs [13,14]. Patients with DM have more necrotic core in coronary atherosclerosis of their native coronary arteries than patients without DM [15-18]. However, the impact of DM on microvascular obstruction and on restenosis after stent implantation into SVGs is not really clear. In type 2 diabetes patients undergoing elective stent implantation into native coronary arteries, the number of microemboli visualized and counted as high-intensity transient signals with a Doppler wire during elective PCI is increased [19]. The incidence of restenosis at 6 months after stent implantation into native coronary arteries is higher in patients with than in those without DM [10]. In contrast, in patients undergoing elective stent implantation into stenotic SVGs, the incidence of no-reflow and of restenosis was similar between those with and without DM [13]. DM is associated with both, systemic inflammation and atherosclerosis [20-22]. Various cytokines and inflammatory mediators (IFN-γ, IL-1, IL-6, TNFα etc.) contribute to the pathogenesis of inflammation observed in atherosclerosis [23-25]. Among these cytokines, TNFα has already been reported to be localized in human atheromatous plaques [26], and to contribute to plaque progression, destabilization, and rupture [27], as well as to progression of restenosis [28].

In the present study, we took advantage of the use of an aspiration device during stent implantation into SVGs and analyzed both, the particulate debris and the soluble vasoconstrictor (catecholamines, endothelin, serotonin, Tx B_2_, tissue factor) and inflammatory mediators (TNFα as a prototype of inflammatory cytokines [29]), in the retrieved coronary aspirate biochemically and by comparison to intravascular ultrasound (IVUS) imaging [30] and to percent diameter stenosis 6 months after stent implantation.

## Methods

### Study cohort

Symptomatic patients with stable angina pectoris and a flow-limiting SVG stenosis (n=40) were recruited. The study was conducted in accordance with the ethical guidelines of the Declaration of Helsinki 1975, and the investigation was approved by the Institutional Review Board (GZ.: 07–3387) and registered at ClinicalTrials.gov (NCT01430884). All patients gave informed consent prior to their inclusion in the study. Using a position statement of the American Diabetes Association on Diagnosis and Classification of DM [31], patients were classified according to their hemoglobin (Hb)A1c-value and their use of antidiabetic medications (with DM: HbA1c ≥ 6.5% and with use of antidiabetic medications *vs*. without DM: HbA1c < 5.7% and without use of antidiabetic medications).

### Quantitative coronary angiography

Patients were on aspirin (100 mg/day) and received 10.000 I.U. heparin intravenously. 17 patients each with and without DM were on clopidogrel (75 mg/day). Coronary angiography was performed using the femoral approach and 6F or 8F guiding catheters. Stenosis severity was quantified using off-line caliper measurements (QCA-MEDIS^R^, Leiden, NL) [32], and thrombolysis in myocardial infarction (TIMI) flow was measured before and after stent implantation [33]. Minimal lumen diameter and reference diameter were determined before, immediately after, and at follow-up 6 months after stent implantation, and the percent diameter stenosis was calculated.

### IVUS and virtual histology (VH) analysis

IVUS was performed before and after stent implantation with a commercially available electronic IVUS catheter (Eagle-EyeTM 20 MHz catheter and R-100 pullback device, Volcano Corporation, Rancho Cordova, CA, USA). The site and the length of the target lesion before stent implantation were retrospectively identified after stent implantation from landmarks in the vascular profile [34,35]. Plaque composition was categorized with VH using a customized software (pcVHTM2.1, Volcano Corp.). All detected plaque components (fibrotic, fibro-fatty, necrotic core, dense calcium) were presented as a fraction of total plaque volume (%) [35].

### Interventional procedure

Implantation of balloon-expandable bare metal stents was performed with direct stenting without prior dilatation/debulking and a stent-to-vessel diameter ratio of 1:1.15, because stenting with predilatation eventually increases plaque mobilisation and debris embolism [36]. To prevent microembolization, a distal balloon occlusion extraction device (GuardWire^R^ Temporary Occlusion & Aspiration System; Medtronic Inc., Minneapolis, MN USA) [37] was used. Before stent implantation, the balloon of the device was inflated at 2 atm with contrast agent. After stent implantation, the catheter with the stent-balloon was removed, and the aspiration catheter was loaded onto the monorail GuardWire^R^. During slow withdrawal of the aspiration catheter, the blood column was retrieved. Then, the balloon was deflated. After PCI patients were loaded with 600 mg of clopidogrel and medication was continued at a dose of 75 mg/day for the next 4 weeks.

### Coronary arterial blood and coronary aspirate

Coronary arterial blood was obtained through the respective aspiration catheter (10 mL into Heparin S-Monovette, SARSTEDT AG & Co, Nümbrecht, Germany) distal to the lesion before stent implantation and served as control. Coronary aspirate (between 10 and 20 mL) was filtered ex vivo through a 40 μm mesh filter. The aspirate dilution by contrast agent was corrected for by reference to the hematocrit. Visible particulate debris was retained on the filter and weighed.

The filtered coronary arterial and aspirate samples were immediately centrifuged (800 g, 10 min, 4°C). Both, particulate debris and plasma samples were quickly frozen in liquid nitrogen and stored at −80°C until further use.

### Total calcium, vasoconstrictors, tissue factor, TNFα, C reactive protein (CRP), and troponin I

Total calcium concentration (sum of ionized and bound/complexed calcium) was measured in coronary arterial and aspirate plasma and in particulate debris after extraction with HCl by atomic absorption spectrophotometry [38].

The serotonin concentration in particulate debris and plasma was measured using an enzyme immunometric assay kit (Assay Designs, Michigan, USA). The TxB_2_ concentration in particulate debris and plasma was determined using the ACE™ enzyme immunoassay (Cayman Chemical Company, Ann Arbor, USA). The TNFα concentration in particulate debris and plasma was determined using a sandwich enzyme immunoassay (Cayman Chemical Company, Ann Arbor, USA). The plasma concentration of endothelin was detected using the immunometric endothelin assay kit (ACE™ enzyme immunoassay, Cayman Chemical Company, Ann Arbor, USA). The plasma concentrations of epinephrine and norepinephrine were determined by HPLC with electrochemical detection (EC 41.000 Chromsystems, München, Germany) using a kit and a reverse phase analytical column (Chromsystems, München, Germany). To determine plasma tissue factor concentration the IMUBIND Tissue Factor Elisa Kit was used, as described by the manufacturer (American diagnostica inc, Stamford, USA).

Peripheral venous blood was taken before and between 6 and 48 h after stent implantation. Serum CRP was determined in peripheral venous blood taken before stent implantation using an immunometric assay kit (ADVIA Clinical Chemistry System, Siemens, Tarrytown, USA). Serum troponin I was measured using a specific 2-side immunoassay detected with the Dimension^R^ RxL Max^R^ Integrated System (Dimension Flex, Dade Behring GmbH, Marburg; and Siemens, Eschborn, Germany) [7,35].

### Vasomotor bioassay

Human coronary arteries and rat mesenteric arteries are characterized by a comparable receptor arrangement for serotonin and TxA_2_[5,7,8]. Therefore, we used rat mesenteric arteries with intact and denuded endothelium (+E/–E). Segments of 2 mm length were mounted in a Mulvany myograph and equilibrated with Krebs-Henseleit buffer. After verification of functionality vessels were incubated with coronary arterial and aspirate plasma, which was diluted to a final ratio of 1:10 vol/vol (after correction for dilution by the hematocrit). Constrictor responses were recorded over 8 min and normalized to the maximum vasoconstriction induced by KCl (% of KCl_max_ =100%) [7,35].

### Statistical analysis

Continuous data are presented as mean±standard error of mean (SEM), categorical data as absolute numbers. Patient characteristics were compared using unpaired *t* test (continuous data) and 2-tailed Fisher’s exact test (categorical data). Mediator concentrations in particulate debris and serum CRP were compared between patients with and without DM using unpaired *t* test. Serum troponin I and TIMI flow grading before and after stent implantation, mediator concentrations in and vasoconstrictor responses to coronary arterial and aspirate plasma, minimal lumen diameter and the percent diameter stenosis before, immediately after and 6 months after stent implantation were compared between patients with and without DM using 2-way repeated measures ANOVA followed by Bonferroni’s post-hoc tests. Linear regression analysis was calculated between the increase in TNFα immediately after stent implantation and angiographic diameter 6 months later in patients with and without DM. All statistics were performed with SPSS Statistics 19.0; SPSS Inc., Chicago, IL, USA. *P*<0.05 was considered significant.

## Results

Patient characteristics (Table 1) and the vessel characteristics (Table 2), respectively, did not differ between the groups with and without DM (apart from their HbA1c-value and antidiabetic medications by definition, body weight and diuretics). TIMI flow was higher after stent implantation, but not different between patients with and without DM. Serum troponin I was increased after stent implantation, but not different between groups (Table 3). Troponin I after stent implantation exceeded the proposed cutoff level of 0.15 μg/L, reflecting myonecrosis [39], in 6 patients with and in 8 without DM.

**Table 1:** Patient characteristics

	**With DM**	**Without DM**	***P*-value**
**demographics**			
number	20	20	1.0
gender, female/male	0/20	0/20	1.0
age [years]	64±2	68±2	0.2
body height [cm]	174±1	173±1	0.5
body weight [kg]	89±2	83±2	**0.04**
**risk factors/comorbidities**			
hypertension	20	20	1.0
BMI [kg/m^2^]	29.4±0.7	27.8±0.6	0.1
smoking	1	3	0.6
hypercholesterolemia	18	20	0.5
family history of CAD	6	5	1.0
**hemodynamics**			
systolic blood pressure [mmHg]	129±6	134±3	0.5
diastolic blood pressure [mmHg]	65±3	66±2	0.6
heart rate [bpm]	66±2	66±3	0.9
**laboratory analysis**			
total cholesterol [mmol/L]	4.5±0.3	4.5±0.2	0.8
HDL cholesterol [mmol/L]	1.0±0.1	1.0±0.1	0.9
LDL cholesterol [mmol/L]	2.5±0.2	2.7±0.2	0.4
CRP [mg/L]	5.6±0.9	4.8±1.0	0.6
triglycerides [mmol/L]	2.7±0.5	1.8±0.3	0.1
serum creatinine [μmol/L]	114.0±6.4	111.8±4.4	0.8
urea nitrogen [mmol/L]	8.0±1.2	7.7±0.6	0.9
eGFR [mL/min/1.73 m^2^]	60.1±2.9	59.7±3.0	0.9
HbA1c [%]	7.7±0.3	5.3±0.2	**< 0.01**
**medication**			
ACE inhibitors	19	15	0.2
AT1-receptor antagonists	3	4	1.0
beta-blockers	19	18	1.0
calcium antagonists	3	1	0.6
statins	19	18	1.0
diuretics	18	10	**0.01**
antidiabetics:	16	0	**< 0.01**
metformin	13	0	**0.05**
glibenclamide	3	0	0.6
insulin	5	0	**0.05**
aspirin	20	20	1.0
clopidogrel	17	17	1.0

Continuous data are presented as mean±SEM, categorical data as absolute numbers. Comparison between patients with and without DM by unpaired *t* test (continuous data) and 2-tailed Fisher’s exact test (categorical data). *ACE* = angiotensin-converting enzyme; *AT1* = angiotensin II type 1; *BMI* = body mass index; bpm = beats per minute; *CAD* = coronary artery disease; *CRP* = C reactive protein; *DM* = diabetes mellitus; *eGFR* = estimated glomerular filtration rate; *HbA1c* = hemoglobin A1c; *HDL* = high density lipoprotein; *LDL* = low density lipoprotein.

**Table 2:** Vessel characteristics

	**With DM**	**Without DM**	***P*-value**
**saphenous vein bypass graft**			
graft-age [years]	9±2	12±1	0.1
**target vessels**			
left anterior descending coronary			
artery	5	6	1.0
left circumflex coronary artery	9	9	1.0
right coronary artery	6	5	1.0
**lesion site**			
ostial	5	3	0.7
proximal	11	13	0.7
distal	4	4	1.0
**lesion with thrombus**	0	0	1.0
**quantitative coronary angiography**			
stenosis diameter [%]	57±2	57±3	0.9
**IVUS-analysis**			
MLA [mm^2^]	3.8±0.3	3.8±0.3	0.9
RLA [mm^2^]	10.2±0.9	10.6±1.0	0.8
plaque burden [%]	69.4±2.2	70.5±2.3	0.7
**stent**			
stent diameter [mm]	3.7±0.1	3.7±0.1	0.6
stent length [mm]	20.4±1.1	23.1±1.3	0.1
**maximal balloon deployment pressure**			
pressure [atm]	20.2±0.8	19.3±0.8	0.4

Continuous data are presented as mean±SEM, categorical data as absolute numbers. Comparison between patients with and without DM by unpaired *t* test (continuous data) and 2-tailed Fisher’s exact test (categorical data). *DM* = diabetes mellitus; *IVUS* = intravascular ultrasound; *LDL* = low density lipoprotein; *MLA* = minimal lumen cross-sectional area in the culprit segment; *RLA* = reference lumen area.

**Table 3:** TIMI flow and serum troponin I before and after stent implantation

	**With DM**		**Without DM**	
	**Before stent implantation**	**After stent implantation**	**Before stent implantation**	**After stent implantation**
**TIMI flow, n = 20 / 20**	2.7 ± 0.1	3.0*	2.8 ± 0.1	3.0*
**troponin I [μg/L], n = 20 / 20**	0.08 ± 0.04	0.32 ± 0.12*	0.04 ± 0.01	0.37 ± 0.12*

Data are mean±SEM. Comparison by 2-way repeated measures ANOVA with Bonferroni’s correction; ^*^P<0.05 before *vs*. after stent implantation. *DM* = diabetes mellitus; *TIMI* = thrombolysis in myocardial infarction.

### Volume and composition of plaques and particulate debris

**Figure 1:**
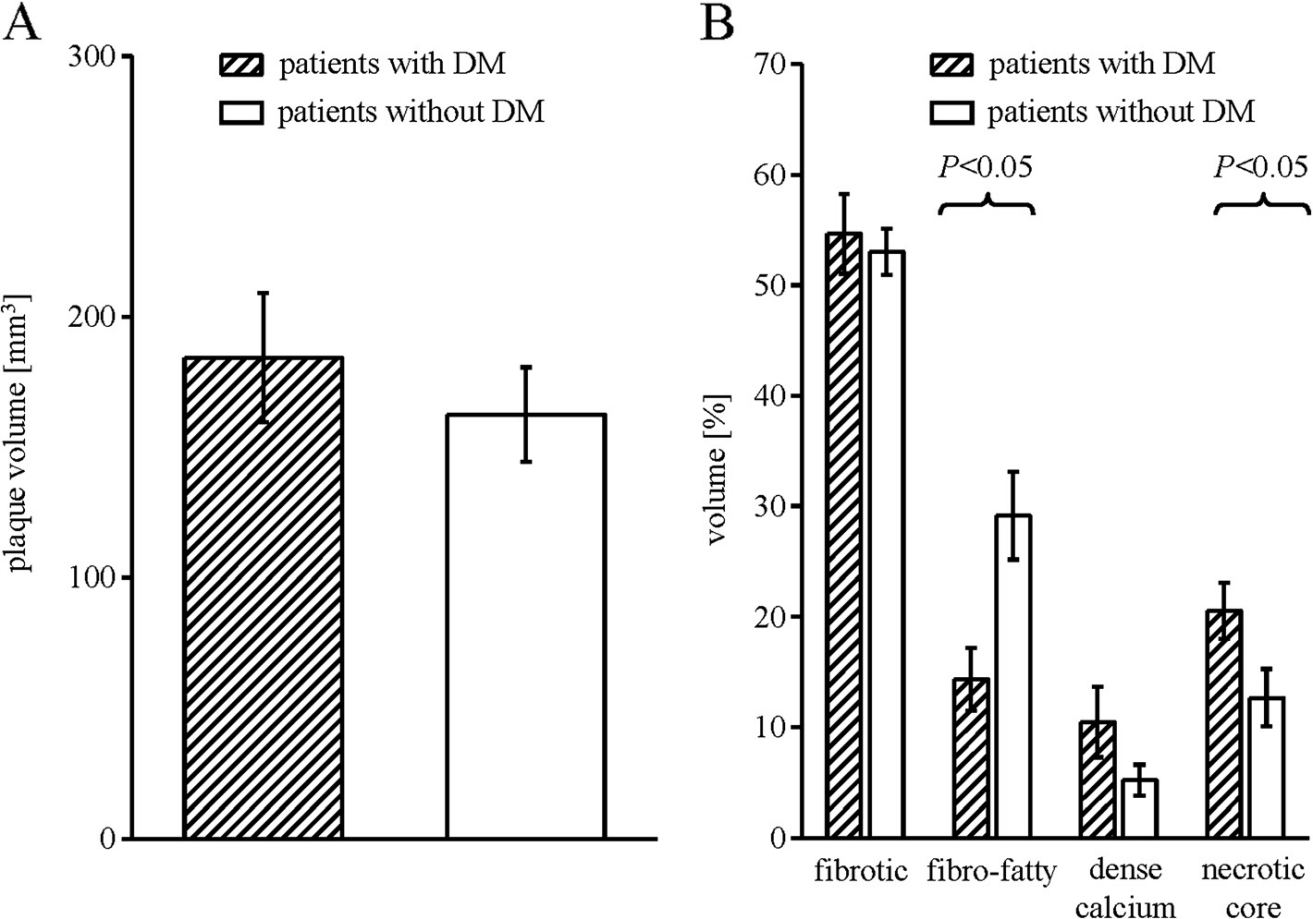
**SVG plaque volume (A) and composition (B) - Data are mean±SEM, comparison between patients with and without DM by unpaired*t *tests. **DM = diabetes mellitus.

**Figure 2:**
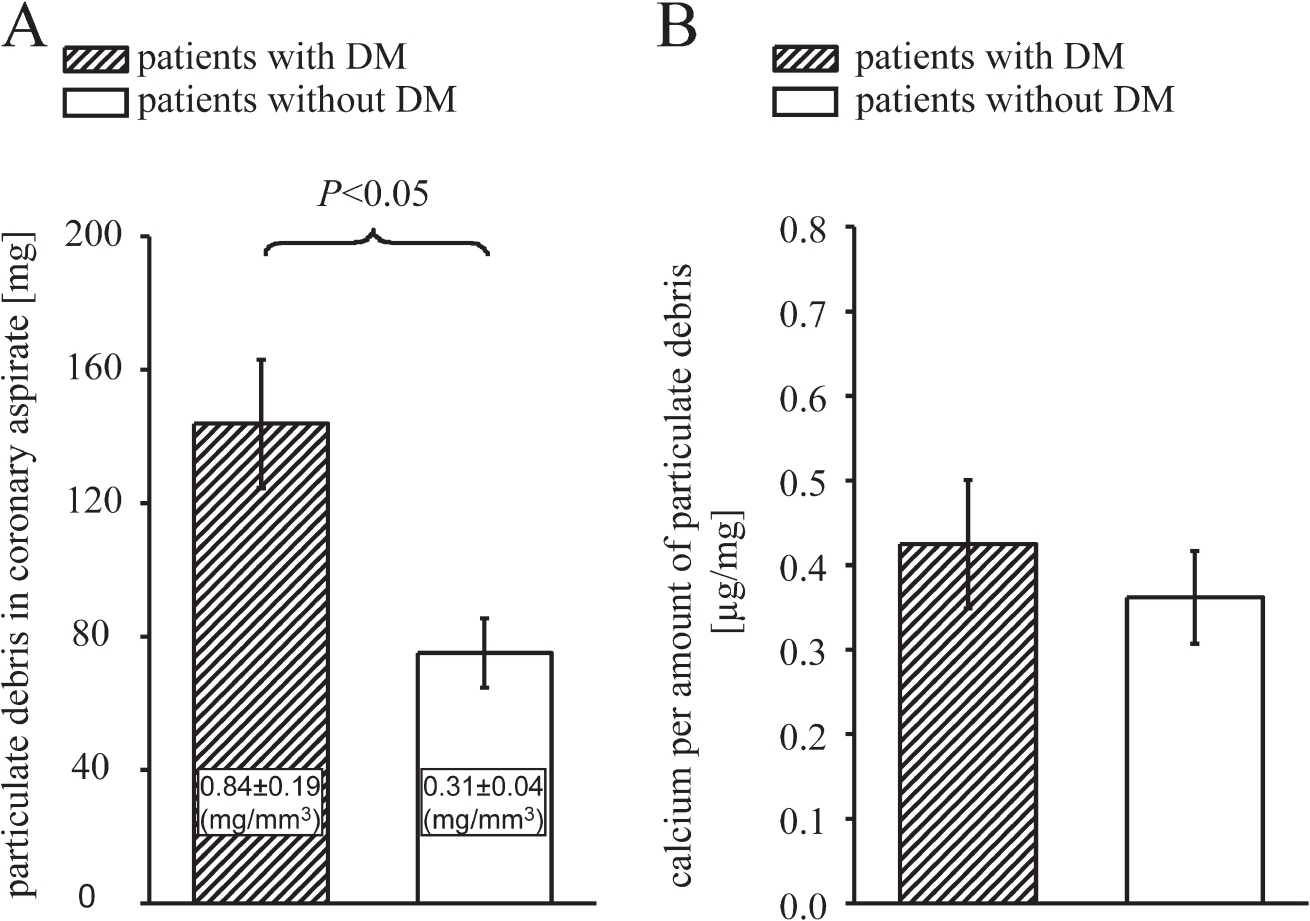
**Amount of released particulate debris (A) and calcium concentration per amount of particulate debris (B) – The normalized amount of released particulate debris to stent volume in numbers in inserts; data are mean±SEM, comparison between patients with and without DM by unpaired*t*tests. **DM = diabetes mellitus.

Plaque volume was comparable between patients with and without DM, but the necrotic core was greater and that of fibro-fatty tissue smaller in patients with DM. (Figures 1A-B). The amount of released particulate debris in coronary aspirate from patients with DM was greater than from those without DM, even when normalized to stent volume, respectively (Figure 2A).

### Total calcium, vasoconstrictors, tissue factor, and TNFα in particulate debris and coronary aspirate plasma and vasoconstrictor action of coronary aspirate plasma

**Figure 3:**
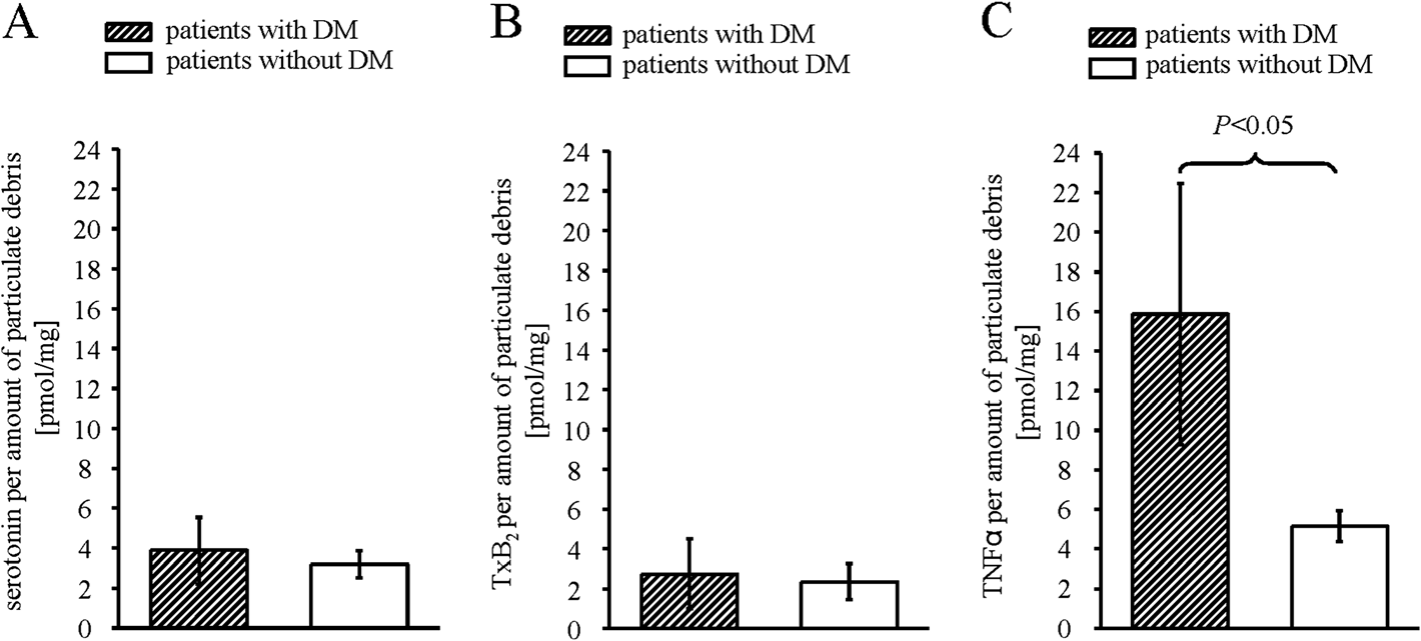
**Concentrations of serotonin (A), TxB**_**2**_**(B), and TNFα (C) per amount of particulate debris- Data are mean±SEM, comparison between patients with and without DM by unpaired*t *tests.** DM = diabetes mellitus; TxB_2_ = thromboxane B_2_, TNFα = tumor necrosis factor α.

Confirming the IVUS VH analysis, the total calcium concentration in particulate debris was not different between patients with and without DM (Figure 2B). The concentrations of serotonin and TxB_2_ in particulate debris did not differ between groups (Figures 3A-B). The concentration of TNFα in particulate debris of patients with DM was higher than in those without DM (Figure 3C).

The total concentration of calcium in coronary aspirate plasma was 2.78±0.14 mmol/L in patients with and 2.39±0.13 mmol/L in those without DM, respectively. The concentrations of endothelin, epinephrine, norepinephrine, and tissue factor in coronary aspirate plasma were not different between patients with and without DM and not altered by stent implantation. The concentrations of serotonin and TxB_2_ in coronary aspirate plasma were increased after stent implantation, but not differently between groups (Table 4). The concentration of TNFα in coronary aspirate plasma was increased after stent implantation in both groups, but more so in patients with than in those without DM (Table 4).

**Table 4:** Baseline and postinterventional concentrations of vasoconstrictors, tissue factor, and TNFα

	**With DM**		**Without DM**	
	**Coronary arterial plasma**	**Coronary aspirate plasma**	**Coronary arterial plasma**	**Coronary aspirate plasma**
**catecholamines, n = 12 / 12**				
**epinephrine [nmol/L]**	0.4 ± 0.1	0.4 ± 0.1	0.4 ± 0.1	0.4 ± 0.1
**norepinephrine [nmol/L]**	3.2 ± 0.8	3.7 ± 0.6	3.4 ± 0.9	4.1 ± 1.3
**endothelin [pmol/L], n = 15 / 16**	1.4 ± 0.6	1.3 ± 0.5	1.9 ± 0.6	1.7 ± 0.6
**serotonin [μmol/L], n = 15 / 16**	0.5 ± 0.2	0.9 ± 0.4^†^	0.4 ± 0.1	1.1 ± 0.5^†^
**thromboxane B**_**2**_**[pmol/L], n = 15 / 16**	46 ± 7	95 ± 9^†^	51 ± 8	82 ± 13^†^
**tissue factor [pmol/L], n = 15 / 16**	8.6 ± 0.5	8.7 ± 0.6	9.6 ± 1.0	8.8 ± 0.8
**TNFα [pmol/L], n = 15 / 16**	1.1 ± 0.3	2.2 ± 0.7^*, †^	0.5 ± 0.2	1.1 ± 0.2^†^

Data are mean±SEM. Comparison by 2-way repeated measures ANOVA with Bonferroni’s correction; ^*^P<0.05 with *vs*. without DM, ^†^*P*<0.05 coronary arterial plasma *vs*. coronary aspirate plasma. *DM* = diabetes mellitus; *TNFα* = tumor necrosis factor α.

**Figure 4:**
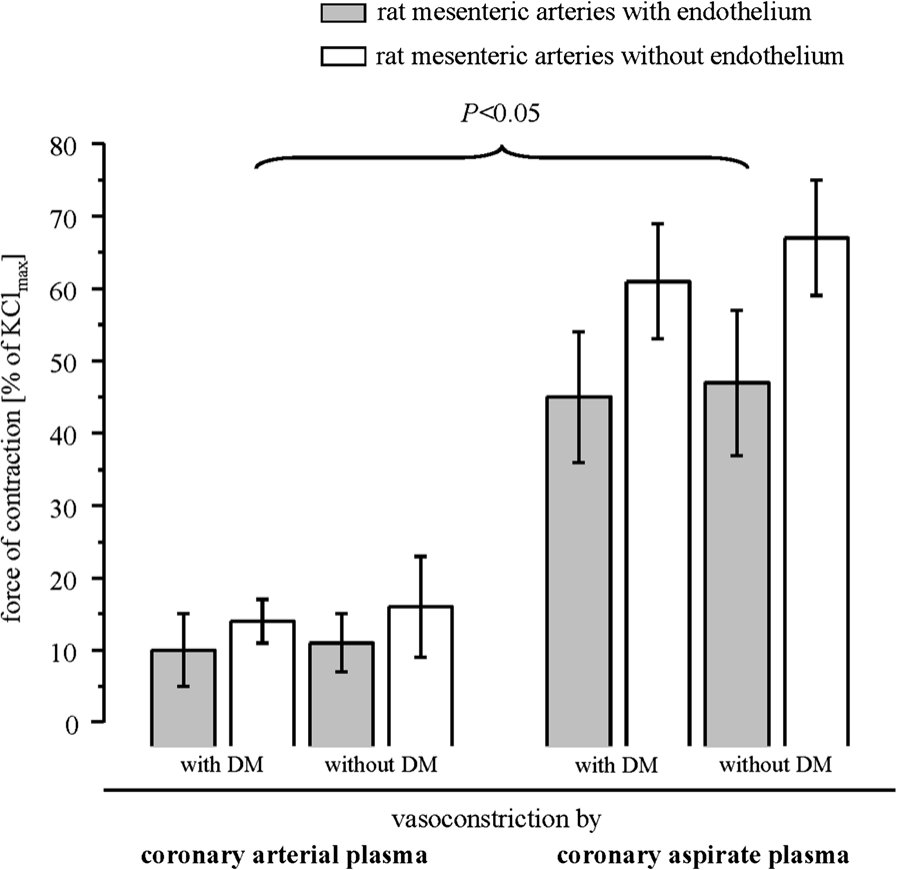
**Vasoconstrictor responses to coronary arterial plasma before and to coronary aspirate plasma after stent implantation – Detected on rat mesenteric arteries with intact endothelium (+E) and denuded -E, respectively.** Data are mean±SEM, comparison between patients with and without DM and before and after stent implantation by 2-way repeated measures ANOVA with Bonferroni’s correction. DM = diabetes mellitus.

As expected from the released soluble vasoconstrictor substances, the coronary aspirate plasma induced comparable vasoconstriction in both groups (Figure 4).

### Angiographic data of percent diameter stenosis at 6 months follow-up

**Figure 5:**
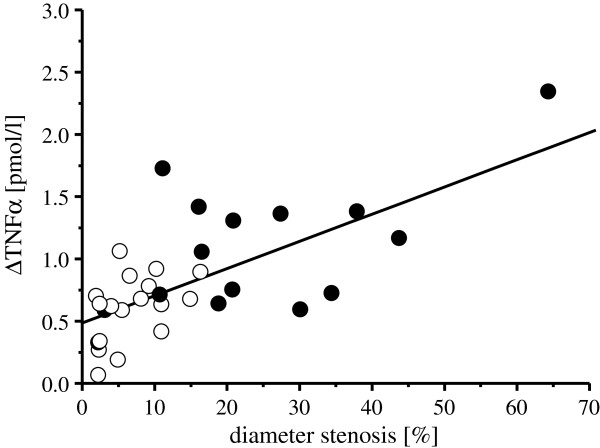
**Correlation between TNFα increase immediately after stent implantation and percent diameter stenosis 6 months later – Linear regression (black line) between the increase in TNFα and percent diameter stenosis in patients with (filled circle) and without (open circle) DM.** TNFα = tumor necrosis factor α.

Before stent implantation, the percent diameter of the stenosis of the SVG did not differ between groups. Immediately after stent implantation, the percent diameter of the stenosis of the SVGs was reduced and not different between groups. Six months after stent implantation, the percent diameter of stenosis of the SVGs was increased in both groups, but more so in patients with than in those without DM (see Table 5). The increase in TNFα immediately after stent implantation correlated with the angiographic diameter reduction 6 months later in patients with and without DM (r=0.69, *P*<0.05; Figure 5).

**Table 5:** Baseline and postinterventional angiographic data of lumen diameter and diameter stenosis

	**With DM**			**Without DM**		
	**Before stent implantation**	**Immediately after stent implantation**	**6 months after stent implantation**	**Before stent implantation**	**Immediately after stent implantation**	**6 months after stent implantation**
**reference lumen diameter [mm]**	2.89±0.20		2.91±0.18	3.33±0.23		3.34±0.23
**minimal lumen diameter [mm]**	1.34±0.10	2.83±0.19^*^	2.22±0.17^†, §^	1.30±0.17^‡^	3.23±0.22^*^	3.15±0.24^‡^
**diameter stenosis [%]**	53.35±1.90	1.80±1.43^*^	22.17±5.22^†, ‡, §^	60.84±4.31	3.01±0.7^*^	6.34±1.11^†, ‡^

Data are mean±SEM. Comparison by 2-way repeated measures ANOVA with Bonferroni’s correction; ^*^P<0.05 before *vs*. immediately after stent implantation, ^†^*P*<0.05 immediately after *vs*. 6 months after stent implantation, ^‡^*P*<0.05 before *vs*. 6 months after stent implantation, ^§^*P*<0.05 with *vs*. without DM; *DM* = diabetes mellitus.

## Discussion

In the present study, graft atherosclerosis of patients with DM was more necrotic and released more particulate debris during stent implantation. Release of the vasoconstrictor substances serotonin and TxB_2_ into the particulate debris and coronary aspirate plasma was comparable between groups and induced a largely comparable vasoconstrictor response *ex vivo*. In contrast, the release of the inflammatory cytokine TNFα into the particulate debris and coronary aspirate plasma was greater in patients with DM, possibly reflecting the greater activity of the underlying atherosclerotic process and associated with greater diameter reduction 6 months after stent implantation.

We have compared a small study cohort of patients with and without DM undergoing elective stent implantation into their stenotic SVGs. We identified more necrotic core in plaque of SVG of patients with DM by IVUS imaging. As expected from the greater volume fraction of necrotic core, the plaque was more unstable [40] and stent implantation induced more particulate debris release in patients with than in those without DM.

Confirming our prior studies [7,8], the concentrations of the vasoconstrictor substances serotonin and TxB_2_ in coronary aspirate plasma were increased after stent implantation in both groups, but not different between groups. Thus, the coronary aspirate also induced a largely comparable vasoconstrictor response ex vivo. The release of serotonin, which is the main coronary vasoconstrictor after stent implantation into SVGs, is attributed to platelet activation during stent implantation. Despite dual inhibition with aspirin and clopidogrel, platelets still release major amounts of serotonin [41]. In the presence of dual platelet inhibition, the release of TxB_2_, which potentiates the vasoconstriction to serotonin [7,8], is not attributed to platelets, but to macrophages in the atherosclerotic vascular wall [42,43], possibly also obscures potential differential diabetics and non-diabetics.

In contrast to serotonin and TxB_2_, the concentration of TNFα in particulate debris and in coronary aspirate plasma was higher in patients with than in those without DM. The release of TNFα is attributed to inflammatory cells in the atherosclerotic vascular wall and associated with plaque remodelling and facilitation of plaque rupture and thrombus formation [44]. TNFα also potentiates the vasoconstriction to serotonin [7,8]. In the present study, we did not detect such a potentiation in vasoconstriction. However, the difference in TNFα levels between the patient groups (with versus without DM) was quite small (1 pmol/L). In our prior study [7], however, we have determined the TNFα-mediated enhancement of vasoconstriction with exogenous application of 25 pmol/l. Prior studies have already confirmed an association between systemic inflammation in atherosclerosis and type 2 diabetes [20-22]. We detected a small, but non-significant difference between patients with and without DM with respect to TNFα-levels in coronary blood before stent implantation, possibly reflecting a difference in systemic inflammation. In line with these arterial TNFα data, peripheral venous serum CRP also tended to be higher. Metformin may have an anti-inflammatory effect by suppressing the production of TNFα [45-47]. In the present study, the treatment with metformin in diabetic patients was stopped before angiography and paused for 48 h. We stratified the TNFα concentrations in coronary arterial and aspirate plasma of diabetic patients with respect to metformin use. The TNFα concentrations in coronary arterial (with metformin: 1.2±0.4 vs. without metformin: 1.1±0.6 pmol/L, n=9/6) and aspirate plasma (with metformin: 2.2±1.1 vs. without metformin: 2.1±0.9 pmol/L, n=9/6) did not differ. We have previously shown that in patients with a severe stenosis in their SVG, the release of TNFα correlates with the incidence of restenosis [6]. In the present study, we confirmed the correlation of the TNFα increase immediately after stent implantation with restenosis 6 months later. In support of this notion, in the present study, patients with DM had a higher TNFα increase immediately after stent implantation and a greater diameter stenosis of their stented SVG at 6 months later than those without DM.

## Conclusion

In conclusion, in patients with DM the greater plaque instability with more particulate debris release appears to account for their greater microvascular obstruction immediately after stent implantation. In the present study, such greater microvascular obstruction, which would be expected from the greater release of particulate debris, was not detected in TIMI flow or troponin I release, reflecting the effective protection with use of the aspiration device. The higher concentration of TNFα in particulate debris and coronary aspirate plasma of patients with DM possibly reflects the activity of the atherosclerotic process and could potentially serve as a biomarker for the incidence and extent of restenosis [6,29].

### Study limitations

Our study is limited to a small number of patients undergoing elective PCI of their SVG and requires prospective confirmation in larger cohorts of patients. Mortality and the incidence of vascular complications are increased in women after SVG stenting [48]. In our cohort including only male patients, we were not able to evaluate gender-specific effects. The model of SVG disease is heterogeneous and also depends on graft age and other factors not related to DM. The plaque composition of SVG differs from that of native vessels [35,49-51]. Nevertheless, as in native coronary arteries, there was also more necrotic core in SVG plaque of patients with DM, as determined by VH based on IVUS imaging before stent implantation. VH has not been validated for use in SVG, and the lack of a clear interface between media and adventitia in SVG makes vessel volume measurements more problematic than in native coronary arteries [35]. However, our most significant finding on the relation of increased aspirate TNFα and restenosis was based on quantitative angiography.

In the present study, we have focused on TNFα as a prototype of inflammatory cytokines. However, also other inflammatory mediators (IFN-γ, IL-1, IL-6) might play a role in the systemic inflammatory process of atherosclerosis in patients with DM, and their levels possibly also correlate with restenosis 6 months after stent implantation.

## Abbreviations

CRP: C reactive protein; DM: Diabetes mellitus; HbA1c: Hemoglobin A1c; IVUS: Intravascular ultrasound; PCI: Percutaneous coronary interventions; SVG: Saphenous vein bypass graft; TIMI: Thrombolysis in myocardial infarction; TNFα: Tumor necrosis factor α; TxA_2_: Thromboxane A_2_; VH: Virtual histology.

## Competing interests

The authors declare that they have no competing interests.

## Authors’ contribution

TB collected patient data, conducted IVUS analyses, performed statistics, drafted the paper. TK enrolled patients and performed interventions. PK and SM enrolled patients, performed interventions and made final comments to manuscript. RE co-designed the study, supervised PCI, made final comments to paper. GH co-designed study, supervised study program, made final comments to paper. PKL designed the study, supervised entire study program, performed biochemical analyses and vasomotor bioassays, finalized the paper. All authors read and approved the final manuscript.

## References

[B1] Heusch G Kleinbongard P Boese D Levkau B Haude M Schulz R Erbel R Coronary microembolization: from bedside to bench and back to bedside Circulation 2009 120 1822 1836 10.1161/CIRCULATIONAHA.109.888784 19884481

[B2] Niccoli G Burzotta F Galiuto L Crea F Myocardial no-reflow in humans J Am Coll Cardiol 2009 54 281 292 10.1016/j.jacc.2009.03.054 19608025

[B3] Dörge H Neumann T Behrends M Skyschally A Schulz R Kasper C Erbel R Heusch G Perfusion-contraction mismatch with coronary microvascular obstruction: role of inflammation Am J Physiol Heart Circ Physiol 2000 279 H2587 H2592 11087208 10.1152/ajpheart.2000.279.6.H2587

[B4] Herrmann J Haude M Lerman A Schulz R Volbracht L Ge J Schmermund A Wieneke H von Birgelen C Eggebrecht H Baumgart D Heusch G Erbel R Abnormal coronary flow velocity reserve following coronary intervention is associated with cardiac marker elevation Circulation 2001 103 2339 2345 10.1161/01.CIR.103.19.2339 11352881

[B5] Leineweber K Boese D Vogelsang M Haude M Erbel R Heusch G Intense vasoconstriction in response to aspirate from stented saphenous vein aortocoronary bypass grafts J Am Coll Cardiol 2006 47 981 986 10.1016/j.jacc.2005.10.053 16516081

[B6] Boese D Leineweber K Konorza T Zahn A Broecker-Preuss M Mann K Haude M Erbel R Heusch G Release of TNF-a during stent implantation into saphenous vein aortocoronary bypass grafts and its relation to plaque extrusion and restenosis Am J Physiol Heart Circ Physiol 2007 292 H2295 H2299 10.1152/ajpheart.01116.2006 17208993

[B7] Kleinbongard P Boese D Baars T Moehlenkamp S Konorza T Schoener S Elter-Schulz M Eggebrecht H Degen H Haude M Levkau B Schulz R Erbel R Heusch G Vasoconstrictor potential of coronary aspirate from patients undergoing stenting of saphenous vein aortocoronary bypass grafts and its pharmacological attenuation Circ Res 2011 108 344 352 10.1161/CIRCRESAHA.110.235713 21183739

[B8] Kleinbongard P Boese D Konorza T Steinhilber F Moehlenkamp S Eggebrecht H Baars T Degen H Haude M Levkau B Erbel R Heusch G Acute vasomotor paralysis and potential downstream effects of paclitaxel from stents implanted for saphenous vein aorto-coronary bypass stenosis Basic Res Cardiol 2011 106 681 689 10.1007/s00395-011-0177-9 21472462

[B9] Quigley PJ Hlatky MA Hinohara T Rendall DS Perez JA Phillips HR Califf RM Stack RS Repeat percutaneous transluminal coronary angioplasty and predictors of recurrent restenosis Am J Cardiol 1989 63 409 413 10.1016/0002-9149(89)90309-3 2521766

[B10] Elezi S Kastrati A Pache J Wehinger A Hadamitzky M Dirschinger J Neumann FJ Schomig A Diabetes mellitus and the clinical and angiographic outcome after coronary stent placement J Am Coll Cardiol 1998 32 1866 1873 10.1016/S0735-1097(98)00467-7 9857865

[B11] Rensing BJ Hermans WR Vos J Tijssen JG Rutch W Danchin N Heyndrickx GR Mast EG Wijns W Serruys PW Luminal narrowing after percutaneous transluminal coronary angioplasty. A study of clinical, procedural, and lesional factors related to long-term angiographic outcome. Coronary Artery Restenosis Prevention on Repeated Thromboxane Antagonism (CARPORT) Study Group Circulation 1993 88 975 985 10.1161/01.CIR.88.3.975 8353925

[B12] Weintraub WS Kosinski AS Brown CL III King SB III Can restenosis after coronary angioplasty be predicted from clinical variables? J Am Coll Cardiol 1993 21 6 14 10.1016/0735-1097(93)90711-9 8417077

[B13] Ahmed JM Hong MK Mehran R Dangas G Mintz GS Pichard AD Satler LF Kent KM Wu H Stone GW Leon MB Influence of diabetes mellitus on early and late clinical outcomes in saphenous vein graft stenting J Am Coll Cardiol 2000 36 1186 1193 10.1016/S0735-1097(00)00861-5 11028469

[B14] Mehta RH Honeycutt E Shaw LK Sketch MH Jr Clinical characteristics associated with poor long-term survival among patients with diabetes mellitus undergoing saphenous vein graft interventions Am Heart J 2008 156 728 735 10.1016/j.ahj.2008.05.033 18926154

[B15] Virmani R Burke AP Kolodgie F Morphological characteristics of coronary atherosclerosis in diabetes mellitus Can J Cardiol 2006 22 Suppl B 81B 84B 10.1016/s0828-282x(06)70991-6 PMC2780829 16498517

[B16] Philipp S Boese D Wijns W Marso SP Schwartz RS Konig A Lerman A Garcia-Garcia HM Serruys PW Erbel R Do systemic risk factors impact invasive findings from virtual histology? Insights from the international virtual histology registry Eur Heart J 2009 31 196 202 19854730 10.1093/eurheartj/ehp428

[B17] Pundziute G Schuijf JD Jukema JW van Werkhoven JM Nucifora G Decramer I Sarno G Vanhoenacker PK Reiber JH Wijns W Bax JJ Type 2 diabetes is associated with more advanced coronary atherosclerosis on multislice computed tomography and virtual histology intravascular ultrasound J Nucl Cardiol 2009 16 376 383 10.1007/s12350-008-9046-9 19437085

[B18] Zheng M Choi SY Tahk SJ Lim HS Yang HM Choi BJ Yoon MH Park JS Hwang GS Shin JH The relationship between volumetric plaque components and classical cardiovascular risk factors and the metabolic syndrome a 3-vessel coronary artery virtual histology-intravascular ultrasound analysis JACC Cardiovasc Interv 2011 4 503 510 21596322 10.1016/j.jcin.2010.12.015

[B19] Otto S Seeber M Fujita B Kretzschmar D Ferrari M Goebel B Figulla HR Poerner TC Microembolization and myonecrosis during elective percutaneous coronary interventions in diabetic patients: an intracoronary Doppler ultrasound study with 2-year clinical follow-up Basic Res Cardiol 2012 107 289 22850870 10.1007/s00395-012-0289-x

[B20] Libby P Inflammation in atherosclerosis Nature 2002 420 868 874 10.1038/nature01323 12490960

[B21] Alexandraki K Piperi C Kalofoutis C Singh J Alaveras A Kalofoutis A Inflammatory process in type 2 diabetes: the role of cytokines Ann N Y Acad Sci 2006 1084 89 117 10.1196/annals.1372.039 17151295

[B22] Packard RR Libby P Inflammation in atherosclerosis: from vascular biology to biomarker discovery and risk prediction Clin Chem 2008 54 24 38 18160725 10.1373/clinchem.2007.097360

[B23] Hartge MM Unger T Kintscher U The endothelium and vascular inflammation in diabetes Diab Vasc Dis Res 2007 4 84 88 17654441 10.3132/dvdr.2007.025

[B24] Goldberg RB Cytokine and cytokine-like inflammation markers, endothelial dysfunction, and imbalanced coagulation in development of diabetes and its complications J Clin Endocrinol Metab 2009 94 3171 3182 10.1210/jc.2008-2534 19509100

[B25] Heusch G Obesity and inflammatory vasculopathy: a surgical solution as ultima ratio? Arterioscler Thromb Vasc Biol 2011 31 1953 1954 10.1161/ATVBAHA.111.232264 21849703

[B26] Tipping PG Hancock WW Production of tumor necrosis factor and interleukin-1 by macrophages from human atheromatous plaques Am J Pathol 1993 142 1721 1728 8506944 PMC1886992

[B27] Waehre T Halvorsen B Damas JK Yndestad A Brosstad F Gullestad L Kjekshus J Froland SS Aukrust P Inflammatory imbalance between IL-10 and TNFalpha in unstable angina potential plaque stabilizing effects of IL-10 Eur J Clin Invest 2002 32 803 810 10.1046/j.1365-2362.2002.01069.x 12423320

[B28] Monraats PS Pires NMM Schepers A Agema WRP Boesten LSM de Vries MR Zwinderman AH de Maat MPM Doevendans PAFM de Winter RJ Tio RA Waltenberger J LM 't H Frants RR Quax PHA van Vlijmen BJM Havekes LM van der Laarse A van der Wall EE Jukema JW Tumor necrosis factor-alpha plays an important role in restenosis development FASEB J 2005 19 1998 2004 10.1096/fj.05-4634com 16319143

[B29] Kleinbongard P Heusch G Schulz R TNFalpha in atherosclerosis, myocardial ischemia/reperfusion and heart failure Pharmacol Ther 2010 127 295 314 10.1016/j.pharmthera.2010.05.002 20621692

[B30] Kleinbongard P Konorza T Boese D Baars T Haude M Erbel R Heusch G Lessons from human coronary aspirate J Mol Cell Cardiol 2011 52 890 896 21762698 10.1016/j.yjmcc.2011.06.022

[B31] American Diabetes Association Diagnosis and classification of diabetes mellitus Diabetes Care 2012 35 S65 S71 10.2337/dc12-0660

[B32] Haude M Caspari G Baumgart D Brennecke R Meyer J Erbel R Comparison of myocardial perfusion reserve before and after coronary balloon predilation and after stent implantation in patients with postangioplasty restenosis Circulation 1996 94 286 297 10.1161/01.CIR.94.3.286 8759068

[B33] TIMI Study Group The Thrombolysis in Myocardial Infarction (TIMI) trial. Phase I findings N Engl J Med 1985 312 932 936 4038784 10.1056/NEJM198504043121437

[B34] Mintz GS Nissen SE Anderson WD Bailey SR Erbel R Fitzgerald PJ Pinto FJ Rosenfield K Siegel RJ Tuzcu EM Yock PG American College of Cardiology Clinical Expert Consensus Document on Standards for Acquisition, Measurement and Reporting of Intravascular Ultrasound Studies (IVUS). A report of the American College of Cardiology Task Force on Clinical Expert Consensus Documents J Am Coll Cardiol 2001 37 1478 1492 10.1016/S0735-1097(01)01175-5 11300468

[B35] Baars T Kleinbongard P Boese D Konorza T Moehlenkamp S Hippler J Erbel R Heusch G Saphenous vein aorto-coronary graft atherosclerosis in patients with chronic kidney disease: more plaque calcification and necrosis, but less vasoconstrictor potential Basic Res Cardiol 2012 107 303 23052640 10.1007/s00395-012-0303-3

[B36] Leborgne L Cheneau E Pichard A Ajani A Pakala R Yazdi H Satler L Kent K Suddath WO Pinnow E Canos D Waksman R Effect of direct stenting on clinical outcome in patients treated with percutaneous coronary intervention on saphenous vein graft Am Heart J 2003 146 501 506 10.1016/S0002-8703(03)00309-0 12947370

[B37] Silber S Albertsson P Aviles FF Camici PG Colombo A Hamm C Jorgensen E Marco J Nordrehaug JE Ruzyllo W Urban P Stone GW Wijns W Guidelines for percutaneous coronary interventions. The Task Force for Percutaneous Coronary Interventions of the European Society of Cardiology Eur Heart J 2005 26 804 847 15769784 10.1093/eurheartj/ehi138

[B38] Zettner A Seligson D Application of atomic absorption spectrophotometry in the determination of calcium in serum Clin Chem 1964 10 869 890 14228268

[B39] Herrmann J Peri-procedural myocardial injury: 2005 update Eur Heart J 2005 26 2493 2519 10.1093/eurheartj/ehi455 16176941

[B40] Boese D von Birgelen C Zhou XY Schmermund A Philipp S Sack S Konorza T Moehlenkamp S Leineweber K Kleinbongard P Wijns W Heusch G Erbel R Impact of atherosclerotic plaque composition on coronary microembolization during percutaneous coronary interventions Basic Res Cardiol 2008 103 587 597 10.1007/s00395-008-0745-9 18787802

[B41] Bax WA Renzenbrink GJ van der Linden EA Zijlstra FJ van Heuven-Nolsen D Fekkes D Bos E Saxena PR Low-dose aspirin inhibits plateled-induced contraction of the human isolated coronary artery. A role for additional 5- hydroxytryptamine receptor antagonism against coronary vasospasm? Circulation 1994 89 623 629 10.1161/01.CIR.89.2.623 8313550

[B42] Fu JY Masferrer JL Seibert K Raz A Needleman P The induction and suppression of prostaglandin H2 synthase (cyclooxygenase) in human monocytes J Biol Chem 1990 265 16737 16740 2120205

[B43] Penglis PS Cleland LG Demasi M Caughey GE James MJ Differential regulation of prostaglandin E2 and thromboxane A2 production in human monocytes: implications for the use of cyclooxygenase inhibitors J Immunol 2000 165 1605 1611 10903770 10.4049/jimmunol.165.3.1605

[B44] Loppnow H Werdan K Buerke M Vascular cells contribute to atherosclerosis by cytokine- and innate-immunity-related inflammatory mechanisms Innate Immun 2008 14 63 87 10.1177/1753425908091246 18713724

[B45] Arai M Uchiba M Komura H Mizuochi Y Harada N Okajima K Metformin, an antidiabetic agent, suppresses the production of tumor necrosis factor and tissue factor by inhibiting early growth response factor-1 expression in human monocytes in vitro J Pharmacol Exp Ther 2010 334 206 213 10.1124/jpet.109.164970 20371705

[B46] Krysiak R Okopien B Lymphocyte-suppressing and systemic anti-inflammatory effects of high-dose metformin in simvastatin-treated patients with impaired fasting glucose Atherosclerosis 2012 225 403 407 10.1016/j.atherosclerosis.2012.09.034 23107042

[B47] Kim SA Choi HC Metformin inhibits inflammatory response via AMPK-PTEN pathway in vascular smooth muscle cells Biochem Biophys Res Commun 2012 425 866 872 10.1016/j.bbrc.2012.07.165 22898050

[B48] Ahmed JM Dangas G Lansky AJ Mehran R Hong MK Mintz GS Pichard AD Satler LF Kent KM Stone GW Leon MB Influence of gender on early and one-year clinical outcomes after saphenous vein graft stenting Am J Cardiol 2001 87 401 405 10.1016/S0002-9149(00)01391-6 11179522

[B49] Mautner SL Mautner GC Hunsberger SA Roberts WC Comparison of composition of atherosclerotic plaques in saphenous veins used as aortocoronary bypass conduits with plaques in native coronary arteries in the same men Am J Cardiol 1992 70 1380 1387 10.1016/0002-9149(92)90285-7 1442604

[B50] Silva JA White CJ Collins TJ Ramee SR Morphologic comparison of atherosclerotic lesions in native coronary arteries and saphenous vein graphs with intracoronary angioscopy in patients with unstable angina Am Heart J 1998 136 156 163 10.1016/S0002-8703(98)70196-6 9665233

[B51] Pregowski J Tyczynski P Mintz GS Kim SW Witkowski A Waksman R Pichard A Satler L Kent K Kalinczuk L Bieganski S Ohlmann P Maehara A Weissman NJ Comparison of ruptured plaques in native coronary arteries and in saphenous vein grafts: an intravascular ultrasound study Am J Cardiol 2006 97 593 597 10.1016/j.amjcard.2005.09.094 16490419

